# Supplementary material for: Use of Cyanobacterium Spirulina (Arthrospira platensis) in Buffalo Feeding: Effect on Mozzarella Cheese Quality
Source: Foods. 2023 Nov 11;12(22):4095. doi: 10.3390/foods12224095 (PMC10670054; doi:10.3390/foods12224095)
Supplement: Supplementary file 1 [file foods-12-04095-s001.zip › foods-2695218-supplementary.pdf]

Table S1. Ingredients (kg/head/day) and chemical composition (% of dry matter, DM, if not otherwise stated) of the experimental total mixed ration.

Item	Dry ration		Lactation ration	
	C group	S group	C group	S group
Ingredients				
Maize silage	6.0	6.0	18.0	18.0
Ryegrass hay	-	-	-	-
Wheat straw	7.0	7.0	3.2	3.2
Alfalfa hay	-	-	2.0	2.0
Ryegrass haylage	5.0	5.0	1.8	1.8
Concentrate mixture	2.0	2.0	8.0	8.0
Spirulina platensis	-	0.05	-	0.1
Chemical composition				
DM, % as fed	57.47	57.55	50.96	51.24
Ash	4.73	4.74	6.54	6.53
CP	9.16	9.35	16.91	17.17
EE	2.48	2.49	5.12	5.12
NDF	60.0	59.76	41.09	40.87
ADF	39.40	39.24	25.15	25.02
ADL	6.06	6.07	4.15	4.17
NFC	23.67	23.65	30.34	30.28
NEL (MJ/kg DM)	4.70	4.68	6.57	6.53

DM, dry matter; CP, crude protein; EE, ether extract; NDF, neutral detergent fiber; ADF, acid detergent fiber; ADL, acid detergent lignin; NFC, non-fibrous carbohydrate; NEL, net energy of lactation.
